# Consideration of pain felt by patients in the ICU

**DOI:** 10.1186/s40560-017-0268-2

**Published:** 2017-12-28

**Authors:** Ryuichi Hasegawa

**Affiliations:** Department of Emergency and Intensive Care Medicine, Mito Clinical Education and Training Center, Tsukuba University Hospital, Mito Kyodo General Hospital, 3-2-7 Miyamachi, Mito, Ibaraki, 310-0015 Japan

**Keywords:** ICU, Pain management, Prognosis

## Abstract

Patients in the ICU are often treated under extreme conditions, with the patient often fearful of losing his life or experiencing severe pain. As a result, high-quality pain management is required. However, response to pain is often inadequate due to continuous administration of sedatives, difficulties in communicating with intubated patients, and/or poor awareness of pain in patients not receiving surgery. Reports on difficulties in pain management in the ICU are many, but few consider the correlation between pain management and patient prognosis. Consequently, consideration on how to implement pain control activities in the ICU to improve patient prognosis is needed.

## Background

Addressing physical and psychological pain experienced by a patient and providing sufficient pain relief should be a top priority of every member of the medical staff, regardless of his/her department. Especially in the ICU, treatment is often provided while the patient is under stress, such as the fear of losing his/her life. Consequently, high-quality pain management with total care is needed [1]. Furthermore, many care and surgical procedures in the ICU are painful and the need to achieve sufficient pain relief by environmental control or analgesics is increased. The harmful effects of poor pain management are well known and are sometimes the result of insufficient knowledge or the lack of institutional commitment [2].

In cases of poor pain control, how is patient outcome, such as survival rate, affected? Pain leads to increased stress on the patient, and stress is known to cause various negative biological reactions such as peptic ulcers, metabolic disorders, catabolic hyperplasia, and immunomodulation [3–7], which could negatively impact patient prognosis. However, we were unable to find any studies which directly investigated the effects of pain on prognosis. One obvious reason for this is that it is ethically problematic to carry out a treatment course that prohibits the use of any analgesics for patients in pain and solely uses sedative drugs. Nonetheless, there are several reports on pain assessment and analgesic procedures for post-operative patients or cancer patients with various complications due to pain, which state that measures to relieve cancer pain improve patient quality of life (QOL), etc. [2, 8]. For example, perioperative analgesia management using epidural anesthesia reduces duration of mechanical ventilation and respiratory complications and leads to improved survival rate of chest trauma patients and a decrease in myocardial ischemic events [9].

## Main body

Although the importance of analgesic management has been pointed out, ICU tends to inadequately respond to pain. The following reasons can be mentioned: (1) continuous intravenous sedation is often administered in many patients with tracheal intubation, and pain assessment cannot be performed properly under such conditions, (2) accurate communication and information acquisition not only regarding the presence of pain but also its location or degree in patients with intubation or central nervous system disease is difficult, and (3) awareness of pain by medical patients who are not subject to a surgical wound or procedure is difficult.

In light of such circumstances, the clinical guideline for pain, analgesia, and delirium in the ICU (PAD guideline) [10] emphasizes the need to consider pain control as a main subject. In the first chapter of the PAD guideline, all members of the ICU medical staff should recognize when a patient feels pain, evaluate the pain, and conduct analgesic management based on standard analgesic protocols. Implementation of this procedure can shorten the time spent in the ICU and improve QOL after discharge by reducing post-traumatic stress disorder (PTSD).

Yamashita et al. retrospectively investigated the status of pain assessment, prognosis of life, and factors related to prognosis, in cases at the ICU of one university hospital [11]. In their report, withholding the use of fentanyl and catecholamine administration was a potential factor of pain, and behavioral pain scale (BPS) > 5 on the pain scale was correlated to an increase in mortality and prolonged ICU stay during mechanical ventilation. This report is quite interesting in that it showed a correlation between strong pain and mortality. BPS > 5 was observed in approx. 20% of ICU patients and the protocol for pain management may have been inadequate, possibly resulting in pain-related stress leading to mortality in those patients.

Other reports also pointed out the possibility that patient stress may increase due to inadequate pain control in the ICU. Bertolini et al. conducted a prospective observational study at 128 ICUs in Italy and showed that 35% of postoperative patients who were not administered any analgesics [12]. In addition, Messerer et al. reported that in pediatric ICU, analgesic drugs were not adequately administered due to concern of side effects [13]. From these reports, difficulty of pain management in the ICU is clearly shown as a deeply rooted problem.

Therefore, consideration of how implementation and standardization of pain control in the ICU could improve patient prognosis is needed in the future. In such a study, contents such as educational activities to disseminate a standard protocol and to promote multi-disciplinary collaboration in the ICU should be included. Recently, several studies have pointed out the benefits of active pain control in neonatal and pediatric care from the same viewpoint as in adult care [13, 14]. At the same time, however, excessive pain relief also carries the potential risk of increased delirium or ventilator dependence with respiratory depression.

## Conclusion

Although pain management in the ICU has some unique difficulties, building a culture that considers pain management to eliminate patient suffering is needed. Sedation-only treatment, without assessment and management of the pain, is not recommended. Similarly, sedation should be kept to a minimum to allow for proper evaluation of pain. Providing aggressive pain control can alleviate patient stress and improve prognosis.

## Abbreviations

BPS: Behavioral pain scale
PAD guideline: The clinical guideline for pain, analgesia, and delirium in the ICU
PTSD: Post-traumatic stress disorder
QOL: Quality of life
